# Selective Phosphonylation of 5′-Adenosine Monophosphate (5′-AMP) via Pyrophosphite [PPi(III)]

**DOI:** 10.1007/s11084-016-9497-y

**Published:** 2016-05-24

**Authors:** Karl Kaye, David E. Bryant, Katie E. R. Marriott, Shohei Ohara, Colin W. G. Fishwick, Terence P. Kee

**Affiliations:** 1 School of Chemistry, University of Leeds, Woodhouse Lane, Leeds, LS2 9JT UK; 2 Chemistry Faculty of Natural & Environmental Sciences, University of Southampton, Highfield Southampton, SO17 1BJ UK; 3 School of Chemistry, University of Glasgow, Joseph Black Building, University Avenue, Glasgow, G12 8QQ UK

**Keywords:** Phosphorus, Prebiotic Chemistry, Origin of Life, Nucleotides

## Abstract

We describe here experiments which demonstrate the selective phospho-transfer from a plausibly prebiotic condensed phosphorus (P) salt, pyrophosphite [H_2_P_2_O_5_^2−^; PPi(III)], to the phosphate group of 5′-adenosine mono phosphate (5′-AMP). We show further that this P-transfer process is accelerated both by divalent metal ions (M^2+^) and by organic co-factors such as acetate (AcO^−^). In this specific case of P-transfer from PPi(III) to 5′-AMP, we show a synergistic enhancement of transfer in the combined presence of M^2+^ & AcO^−^. Isotopic labelling studies demonstrate that hydrolysis of the phosphonylated 5′-AMP, [P(III)P(V)-5′-AMP], proceeds via nuceophilic attack of water at the Pi(III) terminus.

## Introduction

**Fig. 1:**
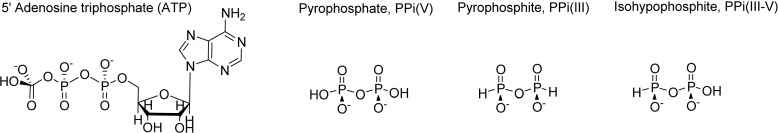
Condensed phosphorus oxyacids used as biological or envisaged as prebiotic energy currency moelcules

Amongst the most important and ubiquitous energy-currency molecules of contemporary biochemistry are activated phosphorus (P) species, condensed polyphosphate esters such as 5′-adenosine triphosphate (5′-ATP; Fig. 1; Harold 1986). These molecules can effectively drive endogonic processes when suitably coupled mechanistically (Harold 1986) subsequently recharging their nucleotide triphosphates via mitochondrial oxidative phosphorylation (Dimroth et al. 2000) and substrate-level phosphorylation (Bochud-Allemann and Schneider 2002). So firmly embedded is ATP in cellular bioenergetics that it is not unreasonable to envisage P-based bioenergetics being amongst the most ancient of biochemical machinery (Baltscheffsky et al. 1999; Serrano et al. 2004, 2007). Pyrophosphate [PPi(V); P_2_O_7_^4−^] has been proposed as a logical ancestor of ATP (Baltscheffsky et al. 1999; Serrano et al. 2004, 2007), but problems persist with its effectiveness to act as a prebiotic phosphorylating agent. Not least of these include the low inherent solubility of this polyanion in the presence of divalent metal ions (Mori et al. 2001) and the fact that phospho-transfer from PPi(V) is very slow in the absence of suitable catalysts (enzymes in the case of contemporary biochemistry; Babich et al. 2012).

We reported recently on a geologically plausible prebiotic ancestor to PPi(V), the closely related condensed P-material, pyrophosphite, PPi(III) [H_2_P_2_O_5_^2−^; Fig. 1; Bryant et al. 2013a, b] and have selected to examine the phosphorus(P)-transfer behaviour of this compound in selected chemical processes of potential value in prebiotic contexts. We envisage PPi(III) to possess a strong prebiotic provenance as this condensed P-compound is found to be readily prepared from H-phosphonate [also called phosphite, Pi(III)] by dehydration under relatively mild conditions (Bryant et al. 2013a, b). We have also recently demonstrated that both Pi(III) and PPi(III) can be readily produced within hot, acidic hydrothermal environments, both lab simulations and in the field at the Hveradalur geothermal site; Kverkfjöll volcanic system, Iceland (Cousins et al. 2013). Amongst such processes are the ability to promote P-transfer leading to both condensed phosphates and organophosphorus species. In our previous paper (Bryant et al. 2013a, b) we described how PPi(III) was capable of phosphonylating phosphate [Pi(V)] in aqueous solution under ambient temperature conditions to afford the mixed-valance condensed P-compound, isohypophosphate, PPi(III-V). We described further how divalent metal ions such as Ca^2+^ and Mg^2+^ had acceleratory effects upon this P-transfer process. Here we expand upon the potential of PPi(III) to function as a P-transfer reagent and report on the selective phosphonylation of 5′-AMP at the 5′-phosphate terminus, mediated by PPi(III). In addition, we ouline how divalent metal ions influence this process, in a similar manner to that observed with PPi(III-V) formation (Bryant et al. 2013a, b) but how also there appear to be synergistic effects in that P-transfer is accelerated when these divalent metal ions are accompanied by carboxylate-containing organic molecules.

## Materials and Methods

### General

Water was purified by ion exchange on a Purite Select Analyst (PSA) reverse osmosis-deionisation system (Purite Ltd., Oxford UK). D_2_O (99.9 % atom D) for NMR analyses and H-phosphonic acid were used as received from Sigma-Aldrich. Isotopically-enriched H_2_^18^O (98.5 %:1.0 %:0.5 % ^18^O:^17^O:^16^O) was purchased from Cambridge Isotope Laboratories). Solution pH measurements were made on a Schochem pH meter calibrated to pH 4 and 7 with commercial (Fisher Chemicals) standards. ^31^P-NMR analyses were performed on a Bruker Avance 500 MHz instrument operating at 202.634 MHz for ^31^P internally referenced to 85 % H_3_PO_4_. Molecular modelling was performed using PC Spartan Pro v1.03. Approximate transition structures were produced using the TS approximation feature within the software. These structures were then optimized using PM3 semi-empirical calculations and a gradient following approach. The resulting transition structures were analyzed using vibrational mode analysis and were characterized as each having a single negative vibrational frequency (shown within Fig. 4).

### Production of Pyrophosphite, Na_2_-PPi(III)

H-Phosphonic acid (16.4 g, 0.2 mols) was dissolved in H_2_O (30 ml). NaOH(s) (8.1 g, 0.2 mols) was added slowly with stirring until all solid had dissolved. The solution was evaporated to dryness under reduced pressure and the residue heated (160 °C) under a dynamic flow of dinitrogen gas for 3 days. A sample was subsequently dissolved in D_2_O for ^31^P-NMR and ^1^H-NMR spectroscopic analysis which revealed a mixture of only starting material and product, PPi(III), pyrophosphite, both as dissociated sodium salts, usually in a 5:95 % ratio respectively. ^1^H-NMR (D_2_O, 27 °C, 300.13 MHz): δ 6.97 (AA’XX’ spin system, ^1^ *J*_PH_ = 666 Hz, ^2^ *J*_PH_ = 9 Hz, ^3^ *J*_HH_ = 8 Hz). ^31^P-NMR (D_2_O, 27 °C, 121.49 MHz): δ -4.98 (AA’XX’ spin system, ^1^ *J*_PH_ = 666 Hz, ^2^ *J*_PH_ = 9 Hz, ^3^ *J*_HH_ = 8 Hz).

### Phosphonylation of 5′-AMP Mediated by Na_2_-PPi(III)

5′-Adenosine monophosphate (5′-AMP) was phosphonylated in the presence of Na_2_-PPi(III) using a procedure modified from that reported (Yamamoto et al. 1988) to afford a range of products which could be identified and quantified by ^31^P-NMR spectroscopy (See SI). Thus, a mixture of Na_2_-PPi(III) (0.29 g, 1.5 mmol) and 5′-AMP (0.037 g, 0.1 mmol) was dissolved in deionized water, the pH of solutions was adjusted to 7 using aqueous NaOH solution (1 M) and the solution made up to 1 mL total volume to arrive at solutions with PPi(III) and 5‘-AMP at 1.5 M and 0.1 M respectively. Solutions were then treated with appropriate additives to achieve the final concentrations as indicated (glycine_,_ G_1_, 0.1 M; diglycine G_2_, 1.0 M; MgCl_2_, 0.1 M; MgCl_2_-G_1_, 0.1-1.0 M; MgCl_2_-G_2_, 0.1-1.0 M) and left to incubate at ambient temperature (*ca*. 20°C) and aliquots removed (0.6 mL) at various time intervals, added to D_2_O for NMR locking purposes (*ca*. 0.1 mL) and reaction progress monitored by ^31^P-NMR spectroscopy at 202.634 MHz operating frequency. ^31^P-NMR (H_2_O; 25°C; pH 7): PPi(III-V)-5‘-AMP: δ -5.5 (dd, ^2^ *J*_PP_ = 20 Hz, ^1^ *J*_PH_ = 666 Hz, Pi(III)]; δ -10.5 [dm, ^2^ *J*_PP_ = 20 Hz, P(V)]. 3‘/2‘-P(III)-5’AMP: δ = 6.1 [dd, ^1^ *J*_PH_ = 652 Hz, ^3^ *J*_PH_ = 10 Hz, Pi(III)]; δ 2.3 [s, br, Pi(V)]**.** 2′/3′-P(III)-5’AMP: δ = 4.9 [ddd, ^1^ *J*_PH_ = 652 Hz, ^3^ *J*_PH_ = 10 Hz, ^3^ *J*_PH_ = 7 Hz, Pi(III)], δ 2.3 [s, br, Pi(V)]. Full measured compositional data for PPi(III), Pi(III), 5′-AMP, PPi(III-V)-5′-AMP, 2′-P(III)-5′-AMP and 3′-P(III)-5′-AMP are collected in accompanying spread-sheet file and were assigned by ^31^P-NMR spectroscopy and by comparison to previously reported data (Yamamoto et al. 1988): *5*’*AMP KK45–52 Collated Results.xlsx*.

### Hydrolysis of PPi(III-V)-5′-AMP Using isoptopically Enriched H_2_^18^O

An aqueous (1 mL) solution containing sodium pyrophosphite, Na_2_-PPi(III), (0.29 g, 1.5 mmol), 5‘-AMP (0.037 g, 0.1 mmol) and MgCl_2_ (0.02 g, 0.1 mmol), was adjusted to pH 7 by slow addition of solid NaOH. This solution was allowed to stand for 24 hrs at ambient temperature before analysis by ^31^P-NMR spectroscopy which reveals the following speciation by integration of peaks: unreacted 5’AMP: 21.5 %; 3′/2′-P(III)-5’AMP: 11.0 % & P(III)P(V)-5‘-AMP: 67.4% (The percentages here refer to only the products of 5’-AMP reaction referenced to total 5’-AMP present. The sum of these products is 5 % of total solution P with 13 % as Pi(III) and unreacted PPi(III) 82 %). This solution was transferred to a micro-distillation apparatus connected to a Schlenk line and the D_2_O removed under reduced pressure. Subsequently, the apparatus was filled with dry dinitrogen gas and H_2_^18^O (1.0 g) added to the residues by syringe. After standing at room temperature for 6 days hydrolysis was found to be complete with the bulk PPi(III) hydrolyzing to Pi(III) and the P(III)P(V)-5′-AMP hydrolyzing back to 5′-AMP. The 3′/2′-P(III)-5’AMP products are relatively more resistant to hydrolysis. Additionally some hydrolysis of the phosphate ester linkage within 5′-AMP generated some Pi(V) which has reacted with PPi(III) to afford PPi(III-V). Excess H_2_^18^O was removed under reduced pressure and replaced with D_2_O (1 mL) and the pH adjusted to 12 via slow addition of solid NaOH. Analysis of the Pi(III) resonances by ^31^P-NMR shows that ^18^O has been incorporated and with a Δδ value of 20 ppb which is consistent with isotopic incorporation.

## Results and Discussion

**Fig. 2:**
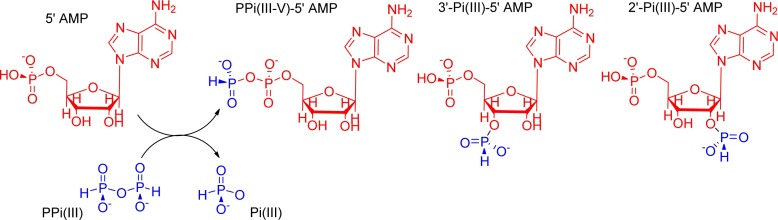
Phosphonylation of 5′-AMP via Na_2_-PPi(III) showing P-transfer to 2′, 3′ & 5′ sites

**Fig. 3:**
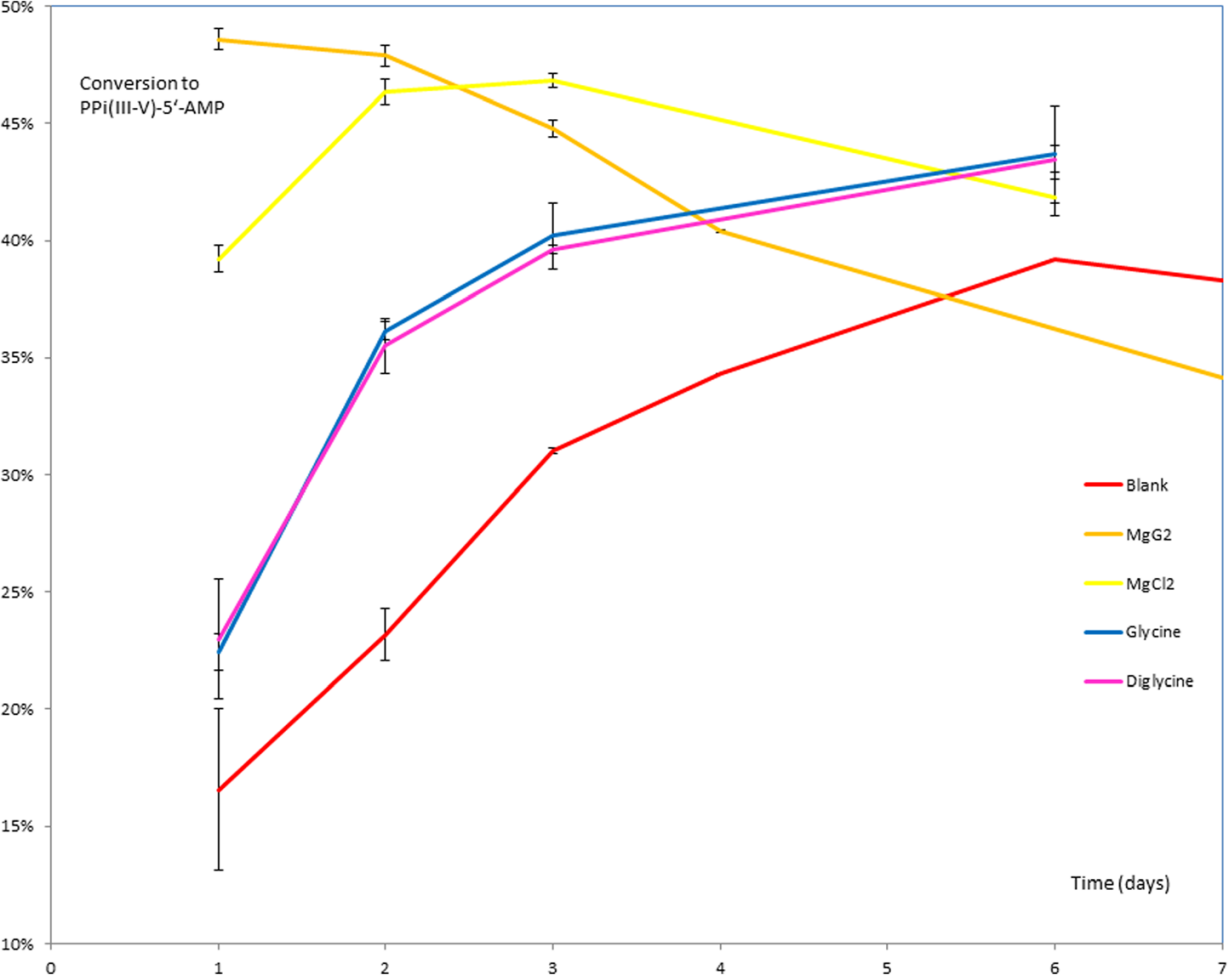
% PPi(III-V)-5′-AMP formation in parallel reactions of PPi(III) (1.5 M) & 5′-AMP (0.1 M), H_2_O, pH 7, *T* = 20 °C control and in the presence of G_1_ (1.0 M), G_2_ (1.0 M), MgCl_2_ (0.1 M) & MgCl_2_-G_2_ (0.1 M; 1.0 M respectively). Error bars reflect data from triplicate runs

In our previously published work on the P-transfer abilities of pyrophosphite, PPi(III) (Bryant et al. 2013a, b) we described how additives could provide a noticable acceleratory effect on product formation. Thus, we wondered to what extent such behaviour might be more widely felt within the general sphere of putatively prebiotic P-transfer chemistry. Thus, aqueous solutions of 5′-AMP (0.1 M) at pH 7 were phosphonylated in the presence of Na_2_-PPi(III) (1.5 M) at ambient temperature (ca. 20 °C) using a procedure modified from Yamamoto (Yamamoto et al. 1988) to afford a range of products, identified and quantified by ^31^P-NMR spectroscopy (vide infra). The major product under these conditions is P-phosphonylated 5'-AMP [PPi(III-V)-5'-AMP; Fig. 2], wherein ca. 16 % of the original 5'-AMP being so phosphonylated after 1 day, rising to ca. 38 % after 6 days (Fig. 3-red control line; 2′ and 3′-functionalised products account for 2–3 % of the total).

**Fig. 4:**
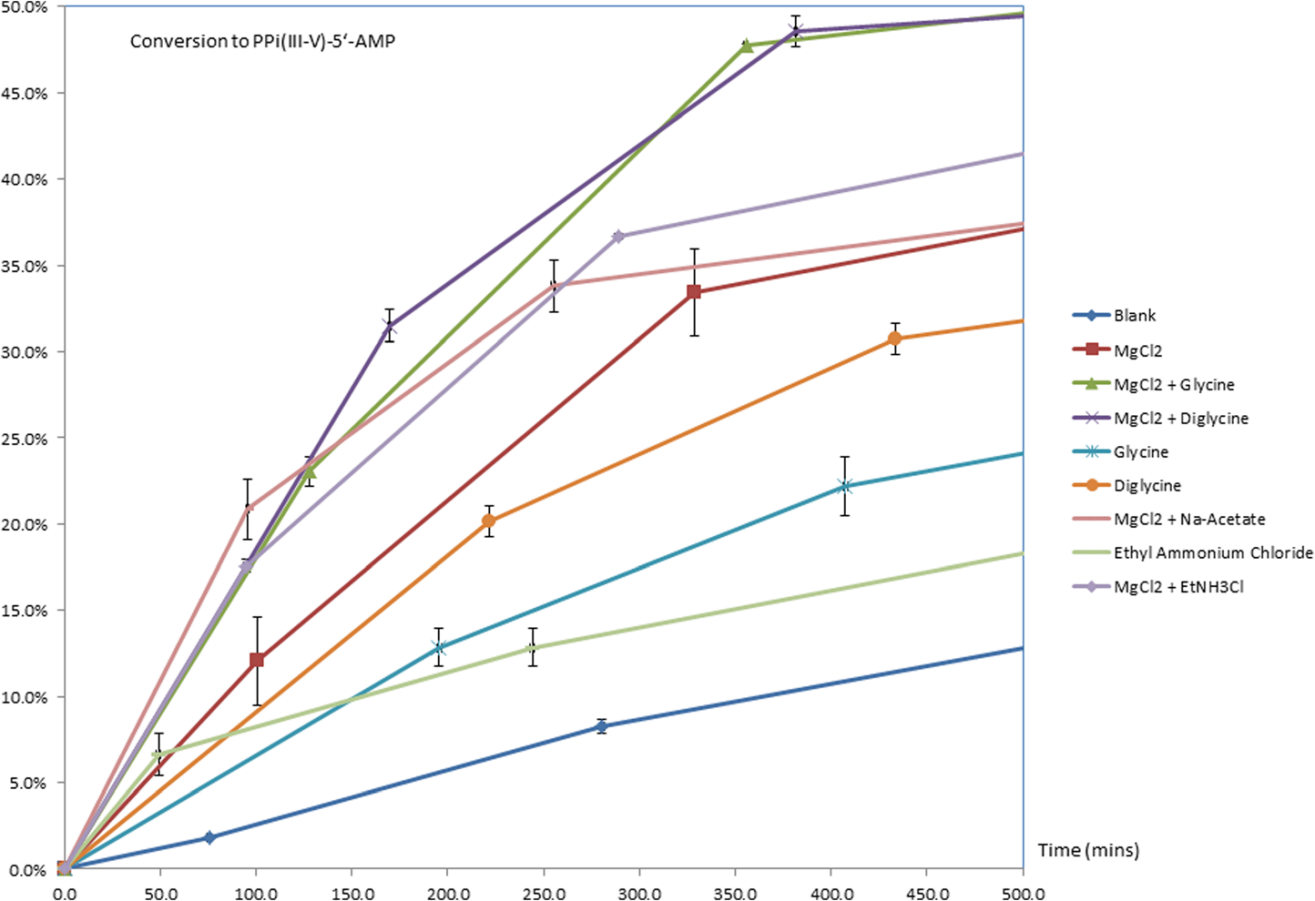
% PPi(III-V)-5′-AMP formation in parallel reactions of PPi(III) (1.5 M) & 5′-AMP (0.1 M), H_2_O, pH 7, *T* = 20 °C control and in the presence of G_1_ (1.0 M), G_2_ (1.0 M), MgCl_2_ (0.1 M); MgCl_2_-G_2_; MgCl_2_ & NaOAc; EtNH_3_Cl and MgCl_2_ & EtNH_3_Cl (0.1 M; 1.0 M respectively). Error bars reflect data from triplicate runs

It is noted that PPi(III-V)-5′-AMP formation is accelerated in the presence of certain additives: MgCl_2_ (0.1 M), CaCl_2_ (0.1 M), KCl (0.1 M), G_1_ (1.0 M), G_2_ (1.0 M) (see attached spreadsheet of raw and processed data: *5*’*AMP KK45–52 Collated Results.xlsx*) from which MgCl_2_ appears to have the most significant effect, affording ca. 2.5 times the amount of PPi(III-V)-5′-AMP after 1 day at 20 °C (Fig. 3-yellow line) against an additive-free control. Even more significant we believe is the observation that adding G_2_ (1.0 M) as a co-factor with MgCl_2_ (0.1 M) further accelerates PPi(III-V)-5′-AMP formation to >3 times after 1 day (Fig. 4-orange line). That this effect is really a synergistic one involving MgCl_2_-peptide and not solely due to the peptide alone is seen by the blue & purple traces (Fig. 3) for PPi(III-V)-5′-AMP production in the presence of G_1_ (1.0 M) and G_2_ (1.0 M) respectively. Both have distinct but significantly more modest acceleratory effects on PPi(III-V)-5′-AMP formation than when combined with MgCl_2_. It can also be seen distinctly from Fig. 3 that both MgCl_2_ and MgCl_2_/G_2_ lead to a decrease in PPi(III-V)-5′-AMP concentration over time when compared to the growth of product over an 8 h period (Fig. 4). This latter graph also shows that EtNH3Cl and NaOAc, as models for the N and C-termini respectively of amino acids, also have acceleratory effects on PPi(III-V)-5′-AMP formation in the presence of MgCl_2_. This is due to hydroylsis of the phosphonylated product (vide infra) which is subject to both general acid and general base catalysis.

**Fig. 5:**
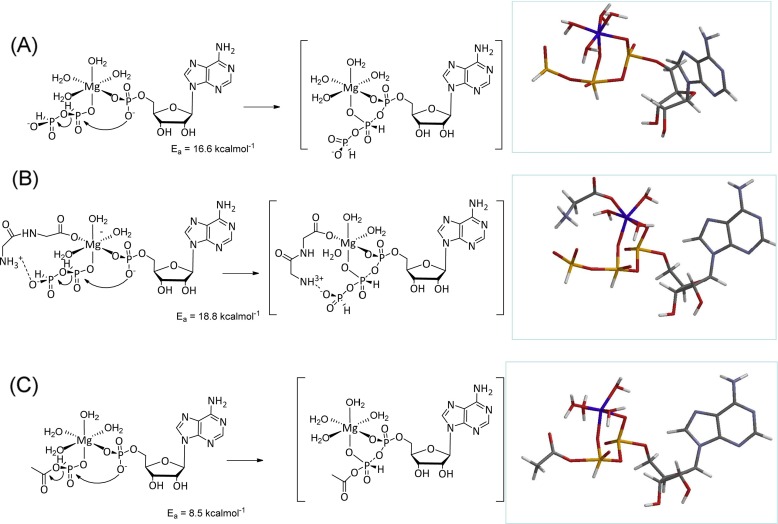
Cartoon models for possible acceleratory effect of combined MgCl_2_-G_2_ co-factors; **a** Mg^2+^ mediated phosphonylation only; **b** G_2_-Mg^2+^ complex model and **c** acylphosphinate intermediate model

Two working models immediately present themselves, based on the proposition that Mg^2+^ acts as a Lewis acid to bring together 5′-AMP and PPi(III) at a common reaction center. Preliminary molecular modeling of such a composite at the PM3 level using the Spartan 1.0.3 (http://www.wavefun.com/products/spartan.html) package, locates a reasonable transition state and returns an activation energy of ca. 16.6 kcalmol^−1^ (Fig. 5a). In the presence of G_1_ or G_2_, two mechanistic possibilities present themselves most clearly. The first model implicates a G_2_-Mg^2+^ carboxylate complex as an intermediate (Fig. 5b) which may have the potential to facilitate [P-O-P] cleavage by stabilizing the leaving phosphite group.

The second model proposes that G_1_ and G_2_ react with PPi(III) to afford acylphosphonates, the key intermediate in PPi(III)-mediated G_1_ coupling. As both 5′-AMP (Bock et al. 1991) and acylphosphonates (Kluger et al. 1975) are known to bind, albeit relatively weakly, to Mg^2+^, binding of both at the same metal centre could facilitate effective phosphonylation of 5′-AMP. An example of this is the simple Spartan PM3 model in which a acetylphosphonate phosphonylates 5′-AMP at a Mg^2+^ centre (Fig. 5c). Whilst not directly comparable to the system reported here we have independent evidence for acylphosphonate formation upon dissolution of Pi(III) in Ac_2_O/AcOH solvent. Both experimental and more detailed computational studies to differentiate between these mechanistic possibilities are currently on-going in our laboratory.

**Fig. 6:**
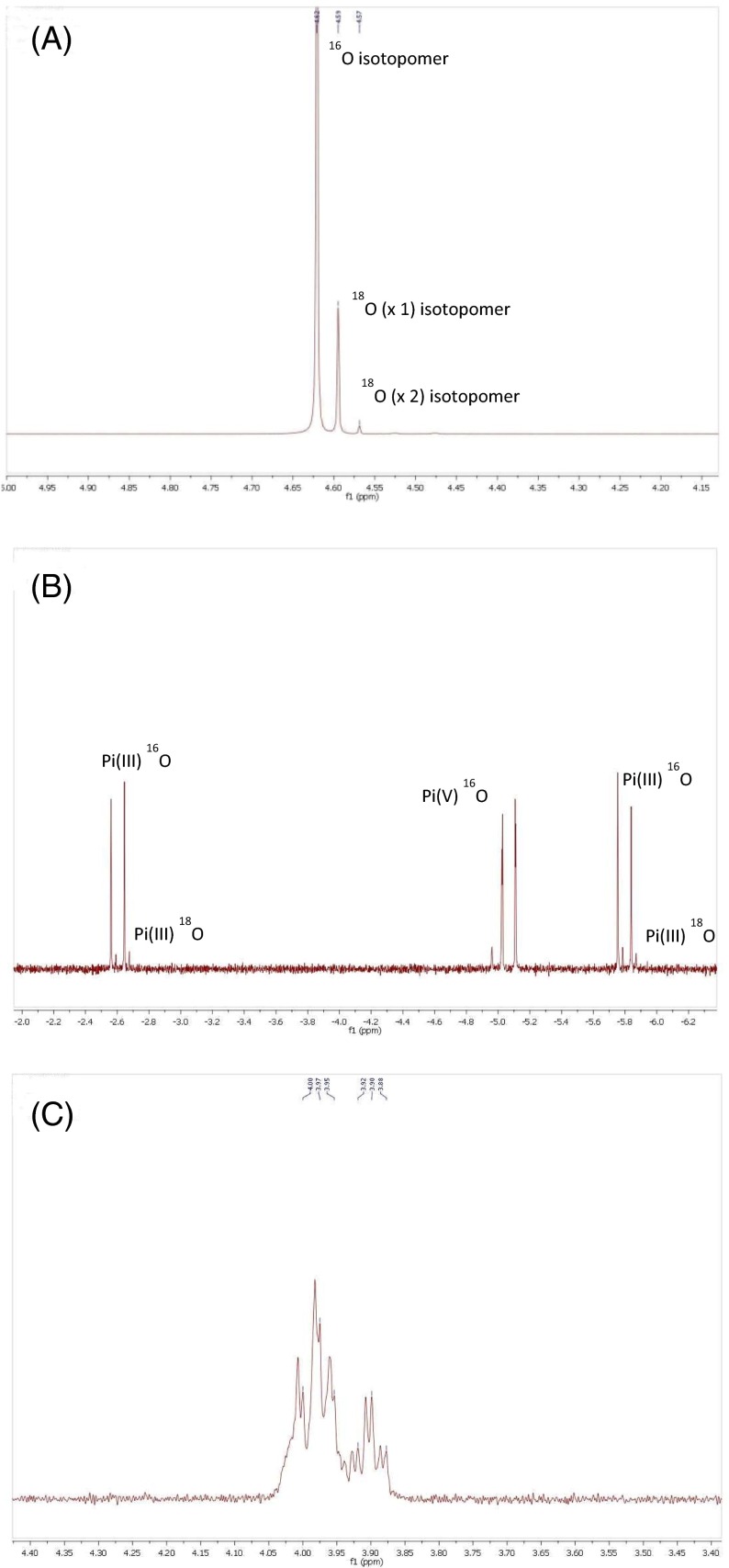
**a** One half of the Pi(III) doublet signal of P(III)P(V)-5‘-AMP in the ^31^P-NMR spectrum (H_2_O; 25°C; 202.634 MHz) emphasizing smaller, satellite signals due to the mono-^18^O and di-^18^O isotopomers. **b** Expansion of the ^31^P-NMR (H_2_O; 25°C; 202.634 MHz) signals for PPi(III-V) in the isotopic exchange experiment on P(III)P(V)-5‘-AMP showing isotopomers associated with the Pi(III) signals but the peak due to Pi(V) (indicated) does not appear to show any ^18^O incorporation. **c** Expansion of the 5’-AMP Pi(V) nucleus signal in the ^31^P-NMR spectrum (H_2_O; 25 °C; 202.634 MHz) after hydrolysis

As illustrated in Fig. 2, PPi(III-V)-5′-AMP, represents a derivatised isohypophosphate, broadly similar to the condensed P-fragment of 5′-ATP. Thus, we considered that the 5′-phosphonylation of 5′-AMP may provide a mechanism for activating the 5′-P(V) moeity towards further functionalisation, most obviously in facilitating the oligomerisation of 5′-AMP. We performed a simple, proof-of-principle isotopic-exchange experiment as to whether this may indeed be feasible; effectively hydrolysing PPi(III-V)-5′-AMP back to 5′-AMP and Pi(III) (the reverse step to that shown in Fig. 2) and ascertaining if ^18^O is incorporated into the Pi(III) or the Pi(V) moiety of 5′-AMP. If the ^18^O was found to be more concentrated in the Pi(V) moiety of 5′-AMP, it would suggest that this Pi(V) moiety had been activated towards hydroysls by having been phosphonylated. If however, the isotopic enrichment was found solely in the Pi(III) groups, then the logical conclusion is phosphonylation of PPi(III-V)-5′-AMP does not activate the Pi(V). Our working model mechanism for isotopic enrichments follows the sequence: (i) PPi(III) is hydrolysed by H_2_^18^O to Pi(III) with ^18^O incorporated; (ii) this *heavy* Pi(III) can then undergo degenerative Pi(III)-exchange via nucleophilic attack at PPi(III) to generate a *heavy* PPi(III); (iii) nucleophilic attack of *heavy* Pi(III) now on *heavy* PPi(III) has a 50 % chance of generating Pi(III) with double ^18^O. The ^31^P-NMR spectroscopy (Fig. 6) shows one half of the Pi(III) doublet signal with smaller, satellite signals due to the mono-^18^O and di-^18^O isotopomers. A separation between isotopomer signals of 20 ppb is typical (Walker et al. 1998) of values expected for the incorporation of one ^18^O atom and the Pi(III) signals above clearly display the ^16^O-isotopomer as dominant but also two further sets of isotopomers with separations, Δδ of 20 and 40 ppb, indicative of incorporation of one and two ^18^O-atoms respectively. The three isotopomers are present in the ratio 71.6:13.7:1.0. Incorporation of ^18^O-isotope is also seen in newly formed PPi(III-V) which we envisage to originate from reaction between PPi(III) and Pi(V) which accrues from the hydrolysis of 5′-AMP when adjusted to pH 12.

The peaks due to the P(III) in PPi(III-V) have isotopomers but the peak due to Pi(V) does not appear to show any ^18^O incorporation (Fig. 6b). Close analysis of the 5′-AMP signal, in the ^31^P-NMR spectrum, after hydrolysis (Fig. 6c) reveals a larger (at δ 3.97) and smaller set (at δ 3.90; ^3^ *J*_PH_ = 4 Hz, ^4^ *J*_PH_ = 2 Hz) of what appears to be triplets of doublets (td). We believe that the difference between the sets of triplets of 70 ppb is too large to be explained as isotopomers of 5‘-AMP (which would indicate incorporation of three ^18^O-atoms) but that the larger td-pattern is due to unreacted 5’-AMP together with the 5′-Pi(V) nucleus of either 3′/2′-P(III)-5’AMP isomers and that the smaller td-pattern is the remaining 3′/2′-P(III)-5’AMP isomer (Fig. 6c).

## Conclusions

Whilst we recognize that many of the concentration ranges and chemical environments used in this study likely map only poorly to early earth geological scenarios (Sleep 2010), much is now known about P-cycling within geological environments (Pasek and Block 2009). We believe this work demonstrates further that condensed P-oxyacids derived from Pi(III) have potential as primitive energy currency molecules. We find that P-transfer from PPi(III) to 5′-AMP appears to be markedly accelerated by divalent cations such as Mg^2+^ and Ca^2+^ and organic co-factors containing acyl-functionalities, scenarios reminiscent of those employed within contemporary biochemistry. The major product of this P-transfer process is the functionalized isohypophosphate, PPi(III-V)-5‘-AMP. In attempting to ascertain whether this condensed P-compound could be considered as an activated form of 5’-AMP, isotopic exchange studies reveal that hydrolysis of PPi(III-V)-5′-AMP via H_2_^18^O takes place preferentially at the Pi(III) rather than Pi(V) terminus, arguing for a greater inherent reactivity at Pi(III). Further studies are continuing to better place some of the above chemistry within putative Hadean geological environments.
